# Clinical impact of Endoscopic Surgical Skill Qualification System (ESSQS) by Japan Society for Endoscopic Surgery (JSES) for laparoscopic distal gastrectomy and low anterior resection based on the National Clinical Database (NCD) registry

**DOI:** 10.1002/ags3.12384

**Published:** 2020-08-31

**Authors:** Tomonori Akagi, Hideki Endo, Masafumi Inomata, Hiroyuki Yamamoto, Toshiyuki Mori, Kazuyuki Kojima, Hiroya Kuroyanagi, Yoshiharu Sakai, Kentaro Nakajima, Hidefumi Shiroshita, Tsuyoshi Etoh, Yoshihisa Saida, Seiichiro Yamamoto, Hirotoshi Hasegawa, Hideki Ueno, Yoshihiro Kakeji, Hiroaki Miyata, Yuko Kitagawa, Masahiko Watanabe

**Affiliations:** ^1^ Gastroenterological and Pediatric Surgery Oita University of Faculty of Medicine Yufu Oita Japan; ^2^ Department of Healthcare Quality Assessment Graduate School of Medicine The University of Tokyo Tokyo Japan; ^3^ Department of Surgery Kyorin University School of Medicine Tokyo Japan; ^4^ First Department of Surgery Dokkyo Medical University Tochigi Japan; ^5^ Department of Gastroenterological Surgery Toranomon Hospital Tokyo Japan; ^6^ Department of Surgery Graduate School of Medicine Kyoto University Kyoto Japan; ^7^ Department of Surgery NTT Medical Center Tokyo Japan; ^8^ Department of Surgery Toho University Ohashi Medical Center Tokyo Japan; ^9^ Department of Surgery Tokai University School of Medicine Kanagawa Japan; ^10^ Department of Surgery Tokyo Dental College Ichikawa General Hospital Chiba Japan; ^11^ Department of Surgery National Defense Medical College Saitama Japan; ^12^ Division of Gastrointestinal Surgery Department of Surgery Kobe University Graduate School of Medicine Kobe Japan; ^13^ Department of Surgery Keio University School of Medicine Tokyo Japan; ^14^ Department of Surgery Kitasato University Kitasato Institute Hospital Tokyo Japan

**Keywords:** endoscopic surgical skill qualification system, laparoscopic distal gastrectomy, laparoscopic low anterior resection, National Clinical Database, short‐term outcome

## Abstract

**Aim:**

This study aimed to evaluate the association between surgeons certified via the Endoscopic Surgical Skill Qualification System (ESSQS) of the Japan Society for Endoscopic Surgery (JSES) and surgical outcomes of laparoscopic distal gastrectomy (LDG) and laparoscopic low anterior resection (LLAR).

**Methods:**

Japanese National Clinical Database data on the patients undergoing LDG and LLAR between 2014‐2016 were analyzed retrospectively. The proportion of cases performed by ESSQS‐certified surgeons was calculated for each procedure, and clinicopathological factors with or without participation of ESSQS‐certified surgeons as an operator were assessed. Then, effects of operations performed by ESSQS‐certified surgeons on short‐term patient outcomes were analyzed using generalized estimating equations logistic regression analysis.

**Results:**

There were 110 610 and 65 717 patients who underwent LDG and LLAR, respectively. The operations performed by ESSQS‐certified surgeons in each procedure totaled 28 467 (35.3%) and 12 866 (31.2%), respectively. A multivariable logistic regression model showed that odds ratios of mortality for LDG and LLAR performed by ESSQS‐certified surgeons were 0.774 (95% CI, 0.566‐1.060, *P* = 0.108) and 0.977 (0.591‐1.301, *P* = 0.514), respectively. Odds ratios for secondary endpoints of anastomotic leakage in LDG and LLAR performed by ESSQS‐certified surgeons were 0.835 (95% CI, 0.723‐0.964, *P* = 0.014) and 0.929 (0.860‐1.003, *P* = 0.059), respectively, whereas that of ileus/bowel obstruction for LLAR performed by ESSQS‐certified surgeons was 1.265 (1.132‐1.415, *P* < 0.001). There were no significant associations between the two operations performed by ESSQS‐certified surgeons and other factors such as mortality and overall complications.

**Conclusions:**

ESSQS certification did not affect postoperative mortality following LDG and LLAR, but annual experience of laparoscopic surgery was associated with it. ESSQS certification may contribute to favorable outcomes regarding anastomotic leakage following LDG and LLAR.

## Graphical abstract

This study aimed to evaluate the association between surgeons certified via the Endoscopic Surgical Skill Qualification System (ESSQS) of the Japan Society for Endoscopic Surgery (JSES) and surgical outcomes of laparoscopic distal gastrectomy (LDG) and laparoscopic low anterior resection (LLAR). The ESSQS contributes to favorable outcomes regarding anastomotic leakage following LDG and LLAR.

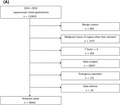

## 1 INTRODUCTION

The use of laparoscopic surgery has continued to increase worldwide ever since laparoscopic distal gastrectomy (LDG) for gastric cancer was introduced in 1994.^1^ As a result of previous large randomized studies, laparoscopic surgery has now become a standard surgical treatment for cStage I gastric cancer.^2^, ^3^, ^4^ Based on other randomized clinical trials, as with LDG, laparoscopic low anterior resection (LLAR) has also spread worldwide.^5^, ^6^, ^7^ The National Survey of Endoscopic Surgery conducted by the Japan Society for Endoscopic Surgery (JSES) found that more than 75% of the low anterior resections performed in Japan in 2017 were done laparoscopically.^8^ It is noteworthy, however, that these important data on the safety and superiority of laparoscopic surgery were mainly obtained from high‐volume centers or hospitals specializing in gastric and rectal surgery. Thus, whether the usefulness and superiority of laparoscopic surgery for gastric and rectal cancer as indicated by these large studies is applicable in general clinical practice remains unclear. To maintain quality and educate trainers, JSES initiated the Endoscopic Surgical Skill Qualification System (ESSQS) in 2004.^9^, ^10^, ^11^ A few small‐scale studies have shown better clinical outcomes when procedures were performed under the supervision of surgeons certified through the ESSQS by JSES.^12^, ^13^, ^14^ However, few reports have evaluated the effectiveness and usefulness of ESSQS with large‐scale data.

The National Clinical Database (NCD) started as a nationwide registry system in 2011 in Japan. Its database has covered more than 95% of surgeries in Japan and has made it possible to investigate the impact of ESSQS on surgical outcomes.

Thus, in the present study, a large‐scale retrospective cohort analysis of patient data in the Japanese NCD database was performed to evaluate the effectiveness of ESSQS by JSES for LDG and LLAR, which have developed into common and standardized operations in terms of lymph node dissection and reconstruction.

## 2 METHODS

### 2.1 ESSQS by JSES

The JSES established the ESSQS and began certification examinations in 2004.^9^, ^10^, ^11^ Two judges assessed unedited videotapes of a procedure in a double‐blinded fashion according to strict common and procedure‐specific criteria. The common criteria were designed to evaluate the surgical set‐up, operator autonomy, display of the surgical field, correct recognition of surgical anatomy, and surgical team co‐operation. Procedure‐specific criteria were used to assess the surgical procedure in a step‐by‐step manner. Of the 1114 surgeons assessed via this qualification system over a 4‐year period, 537 (48.2%) were accredited. To date, the rate of qualification in each surgical field continues to remain at a similar level. During the first year, the inter‐rater agreement of the two judges was low at 0.31, but with revisions of the criteria and the use of consensus meetings, agreement has improved. The surgeons assessed as being qualified by this system experience less frequent complications compared with those who failed the assessment. This system has positively improved and brought standardization to laparoscopic surgery in Japan.^10^, ^11^, ^12^

### 2.2 NCD registration

Details of the registration of data in the Japanese NCD system were described previously.^15^, ^16^ Briefly, the NCD began as a nationwide registry system in Japan that was linked with the surgical board certification system in 2011. As of 2018, over 5000 institutions have participated in this system, with approximately 1 400 000 surgical cases being registered annually.

The Japanese Society of Gastroenterological Surgery (JSGS) designated eight main surgical procedures in the gastroenterological section of the NCD as being especially important in terms of medical standards for improving surgical quality: esophagectomy, distal gastrectomy, total gastrectomy, right hemicolectomy, low anterior resection, hepatectomy, pancreaticoduodenectomy, and surgery for acute diffuse peritonitis. All surgical cases are registered in the NCD, and details including morbidities, comorbidities, postoperative complications, and mortalities are input into the system.

### 2.3 Study population

**FIGURE 1:**
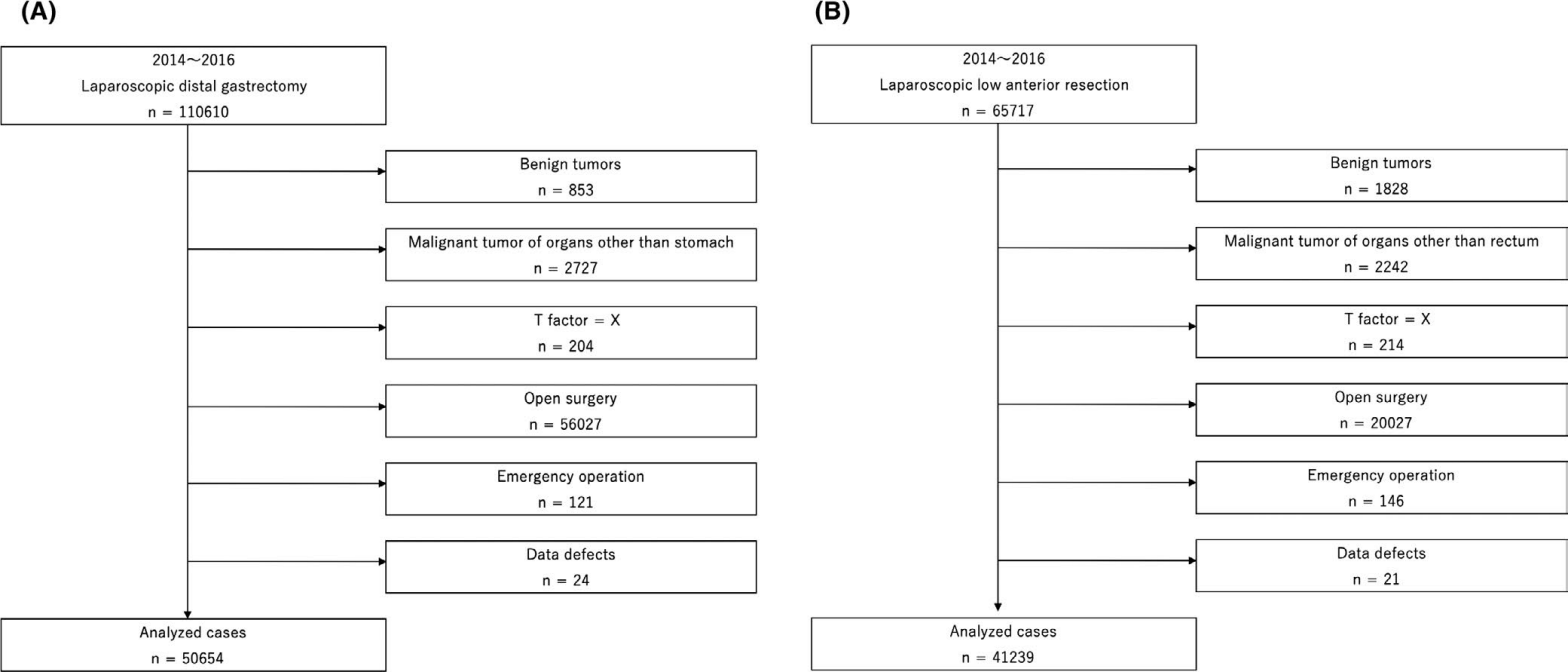
Flowchart detailing the patient selection process in laparoscopic distal gastrectomy (A) and laparoscopic low anterior resection (B)

In total, 110 610 cases of LDG and 65 717 cases of LLAR were registered in the NCD between 2014 and 2016. Among them, benign disease, malignant disease of organs other than the stomach and rectum, open surgery, and emergent surgeries were excluded from this study. Also excluded were 24 cases of LDG and 21 cases of LLAR with data deficits (Figure 1).

### 2.4 Study endpoints

The primary endpoint of the study was mortality (within 30 days after surgery or within 90 days in hospital, respectively). The secondary endpoints included overall postoperative complications (surgical complications: surgical site infection, anastomotic leakage, and pancreatic fistula in LDG; surgical complications: surgical site infection, anastomotic leakage, functional ileus/bowel obstruction, and ileus/bowel obstruction in LLAR; and non‐surgical complications: pneumonia, pulmonary embolism, and urinary tract infection). In particular, anastomotic leakage, pancreatic fistula (Grade B/C), and ileus/bowel obstruction were evaluated as independent complications. An additional endpoint of the study was the incidence of overall postoperative morbidity of ≥grade III according to the Clavien‐Dindo classification,^17^ except for pancreatic fistula. Pancreatic fistula was defined according to the ISGPF (International Study Group of Postoperative Pancreatic Fistula). We also assessed the association of the operations performed by ESSQS‐certified surgeons and laparoscopic operation volumes per year with postoperative complications. To determine a trend in operation volumes in terms of various outcomes, we used cut‐off values that divided the study population into three parts, and we created three groups. Finally, the impact of the operations performed by ESSQS‐certified surgeons on postoperative complications was evaluated by multivariable regression modeling.

### 2.5 Statistical analysis

We used the χ^2^ test for statistical comparisons between groups. We accounted for clustering at the hospital level by use of generalized estimating equations logistic regression analysis for multivariable analysis of the impact of each clinical factor on the short‐term outcomes. The clinical factors included age at surgery (<65, 65 −75, >75 years), sex (male vs female), body mass index (BMI; <18.5, 18.5 < BMI ≤ 25, ≥25 kg/m^2^), preoperative chemotherapy (yes vs no), diabetes mellitus (yes vs no), smoking history (Brinkman index: 0, <400, ≥400), habitual alcohol intake (yes vs no), activities of daily living (no vs any assistance), chronic obstructive pulmonary disease (yes vs no), hypertension (yes vs no), ischemic heart disease (yes vs no), need for preoperative dialysis (yes vs no), previous cerebrovascular disease (yes vs no), chronic steroid use (yes vs no), weight loss (yes vs no), bleeding disorder (yes vs no), preoperative blood transfusion (yes vs no), American Society of Anesthesiologists physical status (ASA‐PS: 1, 2, 3‐5), clinical T stage (T0, Tis, T1a, T1b, T2, T3, T4a, T4b), clinical N stage (N0, N1, N2, N3a, N3b, NX), and clinical M stage (M0, M1). The 7th edition of the American Joint Committee on Cancer TNM classification was used to extract representative tumor depth (T), node metastasis (N), and distant metastasis (M). Additionally, a comparative analysis was conducted between groups in terms of operation time, intraoperative blood loss, and the necessity of transfusion. A two‐sided probability level < 0.05 was considered to indicate a significant difference. The software package R version 3.6.0 (2019; R Foundation for Statistical Computing, Vienna, Austria) was used for statistical analysis. The study protocol was approved by the institutional review board of Oita University (approval number B190275).

## 3 RESULTS

### 3.1 Patient characteristics

Patient and tumor characteristics are shown in Tables 1A and 1B. There were 110 610 and 65 717 patients who underwent LDG and LLAR, respectively. The proportions of participation of ESSQS‐certified surgeons as the operator were 15 341 (30.3%) and 12 866 (31.2%), respectively (Table 1A: Laparoscopic distal gastrectomy).

**TABLE 1A:** Background of patients with laparoscopic distal gastrectomy

	Overall	ESSQS (−)	ESSQS (+)	*P*
n	50654	35313	15341	
Age, years (median [IQR])	69.00 [62.00, 76.00]	70.00 [63.00, 77.00]	69.00 [62.00, 76.00]	<0.001
Age category, years (%)				
<65	15975 (31.5)	10858 (30.7)	5117 (33.4)	<0.001
65‐75	20523 (40.5)	14360 (40.7)	6163 (40.2)	
>75	14156 (27.9)	10095 (28.6)	4061 (26.5)	
Sex (%)				
Female	17621 (34.8)	12321 (34.9)	5300 (34.5)	0.463
Male	33033 (65.2)	22992 (65.1)	10041 (65.5)	
Body mass index (%)				
Normal (18.5 ≤ BMI < 25)	34830 (68.8)	24457 (69.3)	10373 (67.6)	<0.001
Underweight (18.5 > BMI)	5183 (10.2)	3733 (10.6)	1450 (9.5)	
Overweight (25 ≤ BMI)	10641 (21.0)	7123 (20.2)	3518 (22.9)	
Preoperative chemotherapy (%)				
No	50148 (99.0)	35029 (99.2)	15119 (98.6)	<0.001
Yes	506 (1.0)	284 (0.8)	222 (1.4)	
Diabetes mellitus (%)				
No	42158 (83.2)	29333 (83.1)	12825 (83.6)	0.143
Yes	8496 (16.8)	5980 (16.9)	2516 (16.4)	
Brinkman index (%)				
0	27198 (53.7)	19021 (53.9)	8177 (53.3)	0.506
<400	6778 (13.4)	4708 (13.3)	2070 (13.5)	
≥400	16678 (32.9)	11584 (32.8)	5094 (33.2)	
Habitual alcohol intake				
No	23238 (45.9)	16297 (46.2)	6941 (45.2)	0.062
Yes	27416 (54.1)	19016 (53.8)	8400 (54.8)	
COPD (%)				
No	48228 (95.2)	33717 (95.5)	14511 (94.6)	<0.001
Yes	2426 (4.8)	1596 (4.5)	830 (5.4)	
Hypertension (%)				
No	31134 (61.5)	21636 (61.3)	9498 (61.9)	0.175
Yes	19520 (38.5)	13677 (38.7)	5843 (38.1)	
Ischemic heart disease (%)				
No	49024 (96.8)	34146 (96.7)	14878 (97.0)	0.098
Yes	1630 (3.2)	1167 (3.3)	463 (3.0)	
Dialysis (%)				
No	50356 (99.4)	35105 (99.4)	15251 (99.4)	1
Yes	298 (0.6)	208 (0.6)	90 (0.6)	
Cerebrovascular disease (%)				
No	49259 (97.2)	34313 (97.2)	14946 (97.4)	0.111
Yes	1395 (2.8)	1000 (2.8)	395 (2.6)	
Steroid (%)				
No	50162 (99.0)	34962 (99.0)	15200 (99.1)	0.459
Yes	492 (1.0)	351 (1.0)	141 (0.9)	
Weight loss (%)				
No	49781 (98.3)	34690 (98.2)	15091 (98.4)	0.302
Yes	873 (1.7)	623 (1.8)	250 (1.6)	
Bleeding disorder (%)				
No	48819 (96.4)	34041 (96.4)	14778 (96.3)	0.727
Yes	1835 (3.6)	1272 (3.6)	563 (3.7)	
Preoperative transfusion (%)				
No	50302 (99.3)	35064 (99.3)	15238 (99.3)	0.718
Yes	352 (0.7)	249 (0.7)	103 (0.7)	
ASA‐PS (%)				
1	14865 (29.3)	10579 (30.0)	4286 (27.9)	<0.001
2	31636 (62.5)	21775 (61.7)	9861 (64.3)	
3‐5	4153 (8.2)	2959 (8.4)	1194 (7.8)	
T factor (%)				
T0	211 (0.4)	140 (0.4)	71 (0.5)	<0.001
T1a	15130 (29.9)	11040 (31.3)	4090 (26.7)	
T1b	22447 (44.3)	15906 (45.0)	6541 (42.6)	
T2	5765 (11.4)	3796 (10.7)	1969 (12.8)	
T3	4069 (8.0)	2563 (7.3)	1506 (9.8)	
T4a	2464 (4.9)	1474 (4.2)	990 (6.5)	
T4b	199 (0.4)	114 (0.3)	85 (0.6)	
Tis	369 (0.7)	280 (0.8)	89 (0.6)	
N factor (%)				
N0	41824 (82.6)	29641 (83.9)	12183 (79.4)	<0.001
N1	4553 (9.0)	2998 (8.5)	1555 (10.1)	
N2	2487 (4.9)	1561 (4.4)	926 (6.0)	
N3a 7	1291 (2.5)	794 (2.2)	497 (3.2)	
N3b	432 (0.9)	268 (0.8)	164 (1.1)	
NX	67 (0.1)	51 (0.1)	16 (0.1)	
M factor (%)				
M0	50118 (98.9)	34977 (99.0)	15141 (98.7)	<0.001
M1	536 (1.1)	336 (1.0)	200 (1.3)	
Participation of ESSQS‐certified surgeons (%)				
No	35313 (69.7)	—	—	
Yes	15341 (30.3)	—	—	
Participation of JSGS‐certified surgeons (%)				
No	22187 (43.8)	19751 (55.9)	2436 (15.9)	<0.001
Yes	28467 (56.2)	15562 (44.1)	12905 (84.1)	
Laparoscopic operation volume (median [IQR])	26.00 [13.00, 48.00]	21.00 [11.00, 41.00]	40.00 [20.00, 66.00]	<0.001
C1 (1‐17 cases)	18083 (35.7)	14894 (42.2)	3189 (20.8)	<0.001
C2 (18‐41 cases)	16386 (32.3)	11658 (33.0)	4728 (30.8)	
C3 (≥42 cases)	16185 (32.0)	8761 (24.8)	7424 (48.4)	

Abbreviations: ASA‐PS, American Society of Anesthesiologists physical status; BMI, body mass index; COPD, chronic obstructive pulmonary disease; ESSQS, Endoscopic Surgical Skill Qualification System; IQR, interquartile range; JSGS, Japanese Society of Gastroenterological Surgery.

**TABLE 1B:** Background of patients with laparoscopic low anterior resection

	Overall	ESSQS (−)	ESSQS (+)	*P*
n	41239	28373	12866	
Age, yrs (median [IQR])	67.00 [60.00, 74.00]	68.00 [61.00, 75.00]	66.00 [58.00, 73.00]	<0.001
Age category, yrs (%)				
<65	15986 (38.8)	10433 (36.8)	5553 (43.2)	<0.001
65‐75	16577 (40.2)	11565 (40.8)	5012 (39.0)	
>75	8676 (21.0)	6375 (22.5)	2301 (17.9)	
Sex (%)				
Male	14179 (34.4)	9781 (34.5)	4398 (34.2)	0.574
Female	27060 (65.6)	18592 (65.5)	8468 (65.8)	
BMI (%)				
Normal (18.5 ≤ BM I < 25)	27539 (66.8)	18968 (66.9)	8571 (66.6)	0.074
Underweight (18.5> BMI)	4326 (10.5)	3026 (10.7)	1300 (10.1)	
Overweight (25 ≤ BMI)	9374 (22.7)	6379 (22.5)	2995 (23.3)	
Preoperative chemotherapy (%)				
No	38398 (93.1)	26959 (95.0)	11439 (88.9)	<0.001
Yes	2841 (6.9)	1414 (5.0)	1427 (11.1)	
Diabetes mellitus (%)				
No	33825 (82.0)	23149 (81.6)	10676 (83.0)	0.001
Yes	7414 (18.0)	5224 (18.4)	2190 (17.0)	
Brinkman index (%)				
0	22804 (55.3)	16011 (56.4)	6793 (52.8)	<0.001
<400	5568 (13.5)	3645 (12.8)	1923 (14.9)	
≥400	12867 (31.2)	8717 (30.7)	4150 (32.3)	
Habitual alcohol intake				
No	18825 (45.6)	13081 (46.1)	5744 (44.6)	0.006
Yes	22414 (54.4)	15292 (53.9)	7122 (55.4)	
COPD (%)				
No	39744 (96.4)	27343 (96.4)	12401 (96.4)	0.958
Yes	1495 (3.6)	1030 (3.6)	465 (3.6)	
Hypertension (%)				
No	26344 (63.9)	17902 (63.1)	8442 (65.6)	<0.001
Yes	14895 (36.1)	10471 (36.9)	4424 (34.4)	
Ischemic heart disease (%)				
No	40063 (97.1)	27538 (97.1)	12525 (97.3)	0.105
Yes	1176 (2.9)	835 (2.9)	341 (2.7)	
Dialysis (%)				
No	41061 (99.6)	28237 (99.5)	12824 (99.7)	0.035
Yes	178 (0.4)	136 (0.5)	42 (0.3)	
Cerebrovascular disease (%)				
No	40153 (97.4)	27558 (97.1)	12595 (97.9)	<0.001
Yes	1086 (2.6)	815 (2.9)	271 (2.1)	
Steroid (%)				
No	40891 (99.2)	28114 (99.1)	12777 (99.3)	0.027
Yes	348 (0.8)	259 (0.9)	89 (0.7)	
Weight loss (%)				
No	40376 (97.9)	27767 (97.9)	12609 (98.0)	0.383
Yes	863 (2.1)	606 (2.1)	257 (2.0)	
Bleeding disorder (%)				
No	39911 (96.8)	27447 (96.7)	12464 (96.9)	0.477
Yes	1328 (3.2)	926 (3.3)	402 (3.1)	
Preoperative transfusion (%)				
No	40969 (99.3)	28153 (99.2)	12816 (99.6)	<0.001
Yes	270 (0.7)	220 (0.8)	50 (0.4)	
ASA‐PS (%)				
1	12461 (30.2)	8606 (30.3)	3855 (30.0)	<0.001
2	25298 (61.3)	17201 (60.6)	8097 (62.9)	
3‐5	3480 (8.4)	2566 (9.0)	914 (7.1)	
T factor (%)				
T0	217 (0.5)	136 (0.5)	81 (0.6)	<0.001
T1a	8024 (19.5)	5492 (19.4)	2532 (19.7)	
T1b	8405 (20.4)	5793 (20.4)	2612 (20.3)	
T2	18848 (45.7)	12908 (45.5)	5940 (46.2)	
T3	4075 (9.9)	2861 (10.1)	1214 (9.4)	
T4a	859 (2.1)	544 (1.9)	315 (2.4)	
T4b	811 (2.0)	639 (2.3)	172 (1.3)	
Tis	25662 (62.2)	17691 (62.4)	7971 (62.0)	0.042
N factor (%)				
N0	5880 (14.3)	4094 (14.4)	1786 (13.9)	
N1	4461 (10.8)	3073 (10.8)	1388 (10.8)	
N2	122 (0.3)	79 (0.3)	43 (0.3)	
N3a 7	3279 (8.0)	2234 (7.9)	1045 (8.1)	
N3b	1780 (4.3)	1165 (4.1)	615 (4.8)	
NX	55 (0.1)	37 (0.1)	18 (0.1)	
M factor (%)				
M0	38322 (92.9)	26404 (93.1)	11918 (92.6)	0.145
M1a	2292 (5.6)	1535 (5.4)	757 (5.9)	
M1b	625 (1.5)	434 (1.5)	191 (1.5)	
Performance by ESSQS‐certified surgeons (%)				
No	28373 (68.8)	—	—	
Yes	12866 (31.2)	—	—	
Performance by JSGS‐certified surgeons (%)				
No	18720 (45.4)	16823 (59.3)	1897 (14.7)	<0.001
Yes	22519 (54.6)	11550 (40.7)	10969 (85.3)	
Laparoscopic operation volume (median [IQR])	18.00 [10.00, 32.00]	15.00 [8.00, 26.00]	28.00 [17.00, 50.00]	<0.001
C1 (1‐5 cases)	12609 (30.6)	10759 (37.9)	1850 (14.4)	<0.001
C2 (6‐11 cases)	12450 (30.2)	9347 (32.9)	3103 (24.1)	
C3 (≥12 cases‐)	16180 (39.2)	8267 (29.1)	7913 (61.5)	

Abbreviations: ASA‐PS, American Society of Anesthesiologists physical status; BMI, body mass index; COPD, chronic obstructive pulmonary disease; ESSQS, Endoscopic Surgical Skill Qualification System; IQR, interquartile range; JSGS, Japanese Society of Gastroenterological Surgery.

LDG performed by the ESSQS‐certified surgeons group tended to include a higher proportion of patients with high BMI, preoperative chemotherapy, advanced clinical disease stage including clinical T, N, and M, operations performed by JSGS‐certified surgeons, and laparoscopic operation volumes per year (>42 cases). However, the proportion of patients with chronic obstructive pulmonary disease was lower in the non‐ESSQS‐certified surgeons group. In the ESSQS‐certified surgeons group, the proportion of patients with PS 2 was higher and the proportions of patients with PS 1 and PS 3‐5 were lower than those in the non‐ESSQS‐certified surgeons group (Table 1B: Laparoscopic low anterior resection).

LLAR performed by ESSQS‐certified surgeons tended to include a higher proportion of patients with preoperative chemotherapy, high Brinkman index, habitual alcohol intake, advanced clinical disease stage including clinical T and N, operations performed by JSGS‐certified surgeons, and laparoscopic operation volumes per year (>12 cases). However, diabetes mellitus, hypertension, dialysis, cerebrovascular disease, and preoperative transfusion were lower in the patients undergoing surgery performed by the ESSQS‐certified surgeons group than those by the non‐ESSQS‐certified surgeons group. The proportion of patients with PS 2 was higher and the proportions of patients with PS 1 and PS 3‐5 were lower in the ESSQS‐certified surgeons group than those in the non‐ESSQS‐certified surgeons group.

### 3.2 Surgical outcomes

The operative outcomes are shown in Tables 2A and 2B (Table 2A: Laparoscopic distal gastrectomy).

**TABLE 2A:** Surgical outcomes of laparoscopic distal gastrectomy

	Overall	ESSQS (−)	ESSQS (+)	*P*
n	50654	35313	15341	
Operation time, min (median [IQR])	289.00 [239.00, 348.00]	293.00 [243.00, 352.00]	279.00 [230.00, 336.00]	<0.001
Intraoperative blood loss, mL (median [IQR])	33.00 [10.00, 100.00]	40.00 [10.00, 104.00]	25.00 [5.00, 70.00]	<0.001
Intraoperative transfusion (%)				
(−)	49527 (97.8)	34464 (97.6)	15063 (98.2)	<0.001
(+)	1127 (2.2)	849 (2.4)	278 (1.8)	
Overall complications (%)				
(−)	47255 (93.3)	32985 (93.4)	14270 (93.0)	0.112
(+)	3399 (6.7)	2328 (6.6)	1071 (7.0)	
Surgical complication (%)				
(−)	47907 (94.6)	33448 (94.7)	14459 (94.3)	0.034
(+)	2747 (5.4)	1865 (5.3)	882 (5.7)	
Non‐surgical complication (%)				
(−)	49802 (98.3)	34712 (98.3)	15090 (98.4)	0.623
(+)	852 (1.7)	601 (1.7)	251 (1.6)	
Superficial incisional SSI (%)				
(−)	50130 (99.0)	34933 (98.9)	15197 (99.1)	0.175
(+)	524 (1.0)	380 (1.1)	144 (0.9)	
Deep incisional SSI (%)				
(−)	50483 (99.7)	35199 (99.7)	15284 (99.6)	0.432
(+)	171 (0.3)	114 (0.3)	57 (0.4)	
Organ/space SSI (%)				
(−)	49545 (97.8)	34561 (97.9)	14984 (97.7)	0.173
(+)	1109 (2.2)	752 (2.1)	357 (2.3)	
Anastomotic leakage (%)				
(−)	49692 (98.1)	34609 (98.0)	15083 (98.3)	0.02
(+)	962 (1.9)	704 (2.0)	258 (1.7)	
Pancreatic fistula (%)				
(−)	49875 (98.5)	34801 (98.6)	15074 (98.3)	0.016
(+)	779 (1.5)	512 (1.4)	267 (1.7)	
Ileus (%)				
(−)	50402 (99.5)	35146 (99.5)	15256 (99.4)	0.261
(+)	252 (0.5)	167 (0.5)	85 (0.6)	
Bowel obstruction (%)				
(−)	50557 (99.8)	35248 (99.8)	15309 (99.8)	0.639
(+)	97 (0.2)	65 (0.2)	32 (0.2)	
Pneumonia (%)				
(−)	49963 (98.6)	34810 (98.6)	15153 (98.8)	0.083
(+)	691 (1.4)	503 (1.4)	188 (1.2)	
Pulmonary embolism (%)				
(−)	50621 (99.9)	35293 (99.9)	15328 (99.9)	0.342
(+)	33 (0.1)	20 (0.1)	13 (0.1)	
Urinary tract infection (%)				
(−)	50504 (99.7)	35217 (99.7)	15287 (99.6)	0.151
(+)	150 (0.3)	96 (0.3)	54 (0.4)	
Mortality (%)				
(−)	50439 (99.6)	35154 (99.5)	15285 (99.6)	0.2
(+)	215 (0.4)	159 (0.5)	56 (0.4)	

Abbreviations: IQR, interquartile range; SSI, surgical site infection.

**TABLE 2B:** Surgical outcomes of laparoscopic low anterior resection

	Overall	ESSQS (−)	ESSQS (+)	*P*
n	41239	28373	12866	
Operation time, min (median [IQR])	283.00 [224.00, 360.00]	286.00 [227.00, 361.00]	277.00 [217.00, 360.00]	<0.001
Intraoperative blood loss, mL (median [IQR])	30.00 [6.00, 100.00]	30.00 [8.00, 100.00]	20.00 [5.00, 75.00]	<0.001
Intraoperative transfusion (%)				
(−)	40095 (97.2)	27510 (97.0)	12585 (97.8)	<0.001
(+)	1144 (2.8)	863 (3.0)	281 (2.2)	
Overall complications (%)				
(−)	35290 (85.6)	24362 (85.9)	10928 (84.9)	0.014
(+)	5949 (14.4)	4011 (14.1)	1938 (15.1)	
Surgical complication (%)				
(−)	35691 (86.5)	24607 (86.7)	11084 (86.1)	0.115
(+)	5548 (13.5)	3766 (13.3)	1782 (13.9)	
Non‐surgical complication (%)				
(−)	40551 (98.3)	27929 (98.4)	12622 (98.1)	0.017
(+)	688 (1.7)	444 (1.6)	244 (1.9)	
Superficial incisional SSI (%)				
(−)	40372 (97.9)	27757 (97.8)	12615 (98.0)	0.159
(+)	867 (2.1)	616 (2.2)	251 (2.0)	
Deep incisional SSI (%)				
(−)	40890 (99.2)	28111 (99.1)	12779 (99.3)	0.013
(+)	349 (0.8)	262 (0.9)	87 (0.7)	
Organ/space SSI (%)				
(−)	38906 (94.3)	26737 (94.2)	12169 (94.6)	0.162
(+)	2333 (5.7)	1636 (5.8)	697 (5.4)	
Anastomotic leakage (%)				
(−)	37697 (91.4)	25901 (91.3)	11796 (91.7)	0.19
(+)	3542 (8.6)	2472 (8.7)	1070 (8.3)	
Ileus (%)				
(−)	40175 (97.4)	27719 (97.7)	12456 (96.8)	<0.001
(+)	1064 (2.6)	654 (2.3)	410 (3.2)	
Bowel obstruction (%)				
(−)	40833 (99.0)	28098 (99.0)	12735 (99.0)	0.68
(+)	406 (1.0)	275 (1.0)	131 (1.0)	
Pneumonia (%)				
(−)	40986 (99.4)	28178 (99.3)	12808 (99.5)	0.005
(+)	253 (0.6)	195 (0.7)	58 (0.5)	
Pulmonary embolism (%)				
(−)	41195 (99.9)	28346 (99.9)	12849 (99.9)	0.367
(+)	44 (0.1)	27 (0.1)	17 (0.1)	
Urinary tract infection (%)				
(−)	40818 (99.0)	28128 (99.1)	12690 (98.6)	<0.001
(+)	421 (1.0)	245 (0.9)	176 (1.4)	
Mortality (%)				
(−)	41107 (99.7)	28274 (99.7)	12833 (99.7)	0.148
(+)	132 (0.3)	99 (0.3)	33 (0.3)	

Abbreviations: IQR, interquartile range; SSI, surgical site infection.

Operation time, intraoperative blood loss, amount of intraoperative transfusion, and occurrence of anastomotic leakage were significantly lower in the operations performed by ESSQS‐certified surgeons than in those performed by non‐ESSQS‐certified surgeons. The incidences of the surgical complication and pancreatic fistula were significantly more frequent in the operations performed by ESSQS‐certified surgeons than in those performed by non‐ESSQS‐certified surgeons. Mortality was not significantly different between the ESSQ‐ and non‐ESSQ‐certified surgeons groups (0.4% vs 0.5%, *P* = 0.2) (Table 2B: Laparoscopic low anterior resection).

Operation time, intraoperative blood loss, amount of intraoperative transfusion, deep incisional surgical site infection, and pneumonia were significantly lower in the operations performed by ESSQS‐certified surgeons than in those performed by non‐ESSQS‐certified surgeons. In contrast, the incidences of overall complications, non‐surgical complications, ileus/bowel obstruction, and urinary tract infection were significantly more frequent in the operations performed by the ESSQS‐certified surgeons than in those performed by the non‐ESSQS‐certified surgeons. Mortality was not significantly different between the ESSQ‐ and non‐ESSQ‐certified surgeons groups (0.3% vs 0.3%, *P* = 0.148).

### 3.3 Association between the operation performed by ESSQS‐certified surgeons and postoperative complications

(Table 3A: Laparoscopic distal gastrectomy). The multivariable logistic regression model showed that the operations performed by ESSQS‐certified surgeons were an independent predictor of anastomotic leakage. The odds ratio (OR) of anastomotic leakage for the operations performed by the ESSQS‐certified surgeons was 0.835 (95% confidence interval [CI], 0.723‐0.964, *P* = 0.014). There was no correlation between the operations performed by ESSQS‐certified surgeons and mortality. The regression analysis showed that a higher laparoscopic operation volume per year (>42) was an independent predictor of mortality, overall complications, and anastomotic leakage. The ORs of mortality and anastomotic leakage for the higher laparoscopic operation volume per year (>42) group were 0.406 (95% CI, 0.280‐0.588, *P* < 0.001) and 0.777 (95% CI, 0.659‐0.915, *P* = 0.003), respectively. In contrast, the ORs of overall complications and pancreatic fistula for the higher laparoscopic operation volume per year (>42) group were 1.184 (95% CI, 1.084‐1.292, *P* < 0.001) and 1.534 (95% CI, 1.278‐1.840, *P* < 0.001), respectively (Table 3B: Laparoscopic low anterior resection).

**TABLE 3A:** Multivariable analysis of surgical outcomes of laparoscopic distal gastrectomy

	OR	2.5%	97.5%	*P*
*ESSQS +/−*				
Mortality (within 30 d after surgery and within 90 d in hospital)				
ESSQS (−)	Reference			
ESSQS (+)	0.774	0.566	1.060	0.108
Overall complications				
ESSQS (−)	Reference			
ESSQS (+)	1.042	0.967	1.122	0.283
Pancreatic fistula				
ESSQS (−)	Reference			
ESSQS (+)	1.141	0.979	1.330	0.091
Anastomotic leakage				
ESSQS (−)	Reference			
ESSQS (+)	0.835	0.723	0.964	0.014
*Laparoscopic operation volume per year*				
Mortality (within 30 d after surgery and within 90 d in hospital)				
C1 (1‐17 cases)	Reference			
C2 (18‐41 cases)	0.587	0.428	0.806	0.001
C3 (≥42 cases)	0.406	0.280	0.588	0.000
Overall complications				
C1 (1‐17 cases)	Reference			
C2 (18‐41 cases)	1.024	0.939	1.118	0.591
C3 (≥42 cases)	1.184	1.084	1.292	<0.001
Pancreatic fistula				
C1 (1‐17 cases)	Reference			
C2 (18‐41 cases)	1.291	1.072	1.555	0.007
C3 (≥42 cases)	1.534	1.278	1.840	<0.001
Anastomotic leakage				
C1 (1‐17 cases)	Reference			
C2 (18‐41 cases)	0.911	0.781	1.061	0.231
C3 (≥42 cases)	0.777	0.659	0.915	0.003

All models are risk adjusted.

Abbreviation: OR, odds ratio.

**TABLE 3B:** Multivariable analysis of surgical outcomes of laparoscopic low anterior resection

	OR	2.5%	97.5%	*P*
*ESSQS +/−*				
Mortality (within 30 d after surgery and within 90 d in hospital)				
ESSQS (−)	Reference			
ESSQS (+)	0.877	0.591	1.301	0.514
Overall complication				
ESSQS (−)	Reference			
ESSQS (+)	1.028	0.969	1.092	0.362
Anastomotic leakage				
ESSQS (−)	Reference			
ESSQS (+)	0.929	0.860	1.003	0.059
Ileus/bowel obstruction				
ESSQS (−)	Reference			
ESSQS (+)	1.265	1.132	1.415	<0.001
*Laparoscopic operation volume per year*				
Mortality (within 30 d after surgery and within 90 d in hospital)				
C1 (1‐5 cases)	Reference			
C2 (6‐11 cases)	0.762	0.512	1.130	0.180
C3 (≥12 cases)	0.344	0.209	0.565	<0.001
Overall complication				
C1 (1‐5 cases)	Reference			
C2 (6‐11 cases)	1.089	1.012	1.172	0.023
C3 (≥12 cases)	1.113	1.037	1.195	0.003
Anastomotic leakage				
C1 (1‐5 cases)	Reference			
C2 (6‐11 cases)	0.994	0.909	1.087	0.893
C3 (≥12 cases)	0.921	0.844	1.006	0.066
Ileus/bowel obstruction				
C1 (1‐5 cases)	Reference			
C2 (6‐11 cases)	1.214	1.050	1.403	0.009
C3 (≥12 cases)	1.484	1.293	1.702	<0.001

All models are risk adjusted.

Abbreviation: OR, odds ratio.

The multivariable logistic regression model indicated that the operations performed by ESSQS‐certified surgeons showed a tendency for a lower incidence of anastomotic leakage. The OR of anastomotic leakage for the operations performed by ESSQS‐certified surgeons was 0.929 (95% CI, 0.860‐1.003, *P* = 0.059), whereas that of ileus/bowel obstruction was 1.265 (95% CI, 1.132‐1.415, *P* < 0.001). There was no correlation between the operations performed by ESSQS‐certified surgeons and mortality. The regression analysis showed that a higher laparoscopic operation volume per year (>12) was an independent predictor of mortality and anastomotic leakage. The ORs of mortality and anastomotic leakage for the higher laparoscopic operation volume per year (>12) group were 0.344 (95% CI, 0.209‐0.565, *P* < 0.001) and 0.921 (95% CI, 0.844‐1.006, *P* = 0.066), respectively. In contrast, the ORs of overall complications and ileus/bowel obstruction for the higher laparoscopic operation volume per year (>12) group were 1.113 (95% CI, 1.037‐1.195, *P* = 0.003) and 1.484 (95% CI, 1.293‐1.702, *P* < 0.001), respectively. There was a tendency for the level of each OR to be similar between the ESSQS‐certified surgeons group and the laparoscopic operation volume per year (>12) group.

## 4 DISCUSSION

To the best of our knowledge, the present study is the first and largest to investigate the effectiveness of ESSQS for LDG and LLAR through a large‐scale retrospective cohort analysis of patient data in the Japanese NCD. Although the present study did not reveal a contribution of ESSQS to lower mortality, it did show that ESSQS might contribute to a favorable outcome in terms of anastomotic leakage following LDG and LLAR. In terms of the primary endpoint of the present study, the mortality from LDG and LLAR was not significantly affected regardless of whether non‐ESSQ‐ or ESSQ‐certified surgeons performed the procedures. We speculated that the very low mortality rate for LDG and LLAR might have obscured the impact of ESSQ between the two procedures. Although the *P* value for the incidence of anastomotic leakage with LLAR in the ESSQS‐certified surgeons group was 0.059 by multivariate analysis, we thought that at least the ESSQS‐certified surgeons group showed a tendency for a lower incidence of anastomotic leakage, which was considered a meaningful indicator clinically.

In the 8‐year period since the NCD was initiated in 2011, over 5 million cases from more than 4200 facilities have been registered. The efforts of Japanese surgeons have clearly resulted in the successful establishment of this nationwide surgical database. Risk models for 30‐day and operative mortality for several procedures were created, and outcomes for each procedure have been retrospectively published.^18^, ^19^ Additionally, Kanaji et al reported the high quality of the NCD database for gastroenterological surgery in 2019.^20^ During the first audit of the 2015 data, verification of case registration accuracy undertaken on 338 patients (99.4% of extracted cases) showed data accuracy with the maximum number of postoperative variables to be> 95%. The accuracy of mortality and patient status 30 days postoperatively was similarly high at> 99%. The sensitivity was 1.00, and the specificity was 1.00. These results indicated the high accuracy of data entry in the gastroenterological section of the NCD. We analyzed NCD data from more than 250 000 patients registered from 2014 through 2016 in the present study. Many studies have investigated surgeon quality through the evaluation of surrogate markers of superiority such as surgical experience and skill.^21^, ^22^, ^23^, ^24^ From this viewpoint, surgeons certified by ESSQS may be considered to have achieved a certain level of surgical expertise. However, in the past, because of the small number of cases examined, evaluation of the relation between operations performed by ESSQS‐certified surgeons and surgical outcomes has been difficult,^12^, ^13^, ^14^ but the NCD database now makes the investigation of the effect of ESSQS on surgical outcomes possible.

We considered the higher occurrence of ileus/bowel obstruction following LLAR performed by ESSQS‐certified surgeons group and in the laparoscopic operation volume per year (>12) group to be acceptable and natural because of the transitional period for the spread of LLAR, which meant the expansion of indications for highly advanced disease. The background of the patients in the present study showed that more ESSQS‐certified surgeons performed operations for patients undergoing neoadjuvant therapy than those without ESSQS certification. According to the JSES survey, drastic changes in the indication for laparoscopic surgery from early‐stage disease to highly advanced disease, some of which required preoperative treatment, occurred during the period of 2014‐2016.^8^ Although we focused on highly advanced disease and preoperative treatment in the ESSQS‐certified surgeons group, we must recognize that several patient background factors might favorably or unfavorably affect clinical outcomes in the ESSQS‐certified surgeons group such as age, diabetes mellitus, Brinkman index, habitual alcohol intake, cerebrovascular disease, and steroid use, which could not be explained individually. So far, Koh et al have reported that the Centers for Medicare & Medical Services (CMS) high‐stars rating of a hospital did not always signify improved patient outcomes for advanced laparoscopic abdominal operations including gastric cancer and rectal cancer compared to the CMS low‐stars rating.^25^ Additionally, Gambhir et al reported that surgical volume did not appear to be associated with improved patient outcomes owing to the complexity of patients such as those with highly advanced disease and worse general condition through the US News & World Report top ranking for gastroenterology and gastrointestinal operations.^26^ Although leading hospitals such as high‐volume hospitals and the CMS high‐stars hospitals generally might be thought to have better clinical outcomes in terms of all clinical parameters compared to other hospitals, this did not appear to be reflected in clinical practice. In the present study, the operations performed by ESSQS‐certified surgeons correlated significantly with the lower occurrence of the important adverse event of anastomotic leakage. It is considered that anastomotic leakage is associated with basic skill and technique, and ileus/bowel obstruction might result from wider dissection of the peritoneum and lymph nodes, such as pelvic lateral lymph node dissection, for highly advanced disease. Of course, it is important to recognize that there is no data on the level of lymphadenectomy in the NCD database.

Laparoscopic operation volume per year was also a good predictor of postoperative complications such as mortality and anastomotic leakage in LDG and mortality in LLAR. In terms of higher overall complications (*P* < 0.001) and pancreatic fistula (*P* < 0.001) in LDG in institutions treating> 42 patients and higher overall complications (*P* < 0.001) and ileus/bowel obstruction (*P* < 0.001) in LLAR in institutions treating> 12 patients, these unfavorable outcomes showed an almost similar tendency as those in the ESSQS‐certified surgeons group. We speculated that these favorable and unfavorable outcomes might be attributed to the correlation of more ESSQS‐certified surgeons with higher‐volume institutions. In clinical practice, it is reasonable to make clinical decisions based on both the ESSQS and laparoscopic operation volume per year. According to the Japanese Gastric Cancer Association guideline, laparoscopic surgery is recommended for gastric cancer (grade 2, evidence level B), and in the JSCCR (Japanese Society for Cancer of the Colon and Rectum) guideline, laparoscopic surgery for gastric cancer and colorectal cancer (grade 2, evidence level B) is recommended as a standard treatment.^4^, ^27^ Additionally, it is stated that the indication for surgery should be determined at each surgical team level. However, determining the indication for laparoscopic gastrectomy in each hospital is nonobjective and difficult. Therefore, the outcomes shown in the present study could help surgeons to determine the indications for laparoscopic surgery in each hospital because there are no criteria for the evaluation of surgical team level. An operation performed by ESSQS‐certified surgeons is one of the most valuable predictors of optimal clinical outcome in terms of anastomotic leakage.

There are several limitations in the present study. First, it is a retrospective, observational study. Potential bias due to heterogeneity of the surgical quality or performance by each hospital cannot be excluded. Second, this analysis did not overcome all of the uncertainties associated with the details of the surgical procedures, such as the degree of lymphadenectomy, methods or technique of reconstruction, type of approach (e.g. robotic surgery, single‐port surgery, or transanal total mesorectal excision), or types of energy devices used. Third, the oncological and long‐term outcomes are not available from the Japanese NCD. Only data regarding baseline characteristics and short‐term outcomes were allowed to be input. A definitive conclusion as to the oncological validity of this surgery must also depend on the data from other clinical trials. Fourth, the clinical impact of operations in which ESSQS‐certified surgeons participated as an assistant could not be evaluated.

In conclusion, ESSQS certification did not affect postoperative mortality following LDG and LLAR, but annual experience of laparoscopic surgery was associated with it. ESSQS certification may contribute to favorable outcomes regarding anastomotic leakage following LDG and LLAR.

## DISCLOSURE

Funding: Author YK received lecture fees and was supported by a grant from TAIHO PHARMACEUTICAL CO, LTD. and CHUGAI PHARMACEUTICAL CO, LTD. Authors HE, HY, and HM were supported by grants from the National Clinical Database, Johnson & Johnson K.K., and Nipro Co.

Conflict of Interest: Authors declare no conflicts of interest for this article.
